# Priorities in the Polish health care system

**DOI:** 10.1007/s10198-016-0831-0

**Published:** 2016-09-28

**Authors:** Sylwia Nieszporska

**Affiliations:** Faculty of Management, Czestochowa University of Technology, Armii Krajowej 19B, 42-200 Częstochowa, Poland

**Keywords:** Polish health care, Public expenditures, Goals of a system, Health policy

## Abstract

Since 1999, Polish health policy has changed, the quality of services has increased, and also the level of financing, mainly from public benefits. Despite constant growth of indexes reflecting the health status of Polish society, such as life expectancy, quality of life, or decreasing index of deaths at birth, just as in the majority of European countries, in Poland the society is growing older, which implies the necessity to reorganize the system. In this paper, the author has described the most important factors that determine the operation of the health system in Poland, as well as presents the ways it was restructured over the last few years, taking into consideration the structural, legislative, financial, organizational, and quantitative aspects. Also, the latest trends in Polish health policy, which take into account new goals of the system, have been presented within.

## Introduction

The Polish health care system, just as any other, is deeply rooted in the economic reality of the country, and although, referring to the human development index, according to which Poland currently belongs to highly developed countries, its health care system still feels the consequences of historical turmoil and the political experiences of the country. Setbacks and tremendous negligence in the scope of health care, which took place after the Second World War, seem to impact a series of problems in the sector, which are still felt nowadays. Moreover, a specific model of human behavior and the current hierarchy system, so much suppressed or even misshaped in the second half of the 20th century in Poland, have resulted in numerous consequences in the areas such as professional ethics of doctors, mutual patient-doctor relationships, or widely understood sector management.

At present, the Polish health care system, although established within the framework created by the EU restrictions, is still strongly influenced by the internal politics of the state. Most health care institutions are public and the monopolized public purse, from the position of the centralized body, is not able to satisfy the demand for health services and does not notice various needs and preferences of patients in different regions of the country.

## Polish health care system

Health care services in Poland have been financed, supervised, and controlled by the Ministry of Health, the National Health Fund (NHF) and local governments since 2003.

The Ministry of Health plays a key role in determining the health policy of the country, financing and implementing health programmes, financing some highly specialized services, scientific research, and educating medical staff. It also fulfils numerous supervisory and management functions with relation to some institutions of the health care system, including the National Health Fund—a public purse.

The role of founding bodies of most of public health care institutions are local governments, which are also responsible for identifying health needs of their citizens, planning the supply of health services, and promoting health within their territories.

The legal frameworks of the current Polish health care system have been determined by numerous laws and regulations, the most important of them nowadays seem to be: the law on health care institutions (April 15, 2011) [12] and the law on health care services financed from public resources (September 27, 2004) [11]. The first one determines primarily the conditions of providing and the scope of health care services financed from public resources, responsibilities of public authorities in the scope of ensuring equal access to services and the principle of common—obligatory and voluntary health insurance. It has become possible to transform independent public health care institutions into commercial law partnerships and create new entities within this legal status.

The place where a patient first contacts the health care system is Basic Health care Units (BHU), where the patient, by receiving a proper referral, can obtain access to specialized health care. Out-patient care (basic and specialized) is conducted by health care entities such as out-patient clinics, dispensaries, and doctor’s practices, which largely remain in private hands.

## Financing of Polish health care system

Apart from the public purse, the source of health care financing in Poland has been the national budget and local governments budgets. Over the years, the structure of these expenditures has changed slightly. Since 1999, expenditures from the national budget have decreased substantially, while the ones coming from local governments have grown. However, their general level is still almost two times lower than the expenditures of Scandinavian countries [5], or by about 7 % points lower than expenditures in Germany [8] and in 2014 was equal 4.5 % GDP.

Most of the public expenditure is designated to treatment taking place in Polish hospitals (43 %), which calculated per capita ($400) constitutes the amount that placed Poland in 2011 relatively low among the EU countries that belong to the OECD [13].

While the level of health sector financing from public resources is one of the lowest in Europe, the level of financing of the sector from private resources does not come as a big surprise. The health needs of 38.5 million Polish citizens who lived in the country in 2012 (Central Statistical Office) and the failure of public financing determined that the majority of this expenses of this goal coming from the private resources of households (23 % of all expenditures on the health care sector constituted private expenditures [13]. The majority of private expenditures of households (as much as 68 % in 2013) were dedicated to medicaments, 29 % to outpatient health care, and 2.3 % to hospital treatment [4].

## Health condition of Polish citizens

The situation of Polish citizens with regard to the most common health status measure, which is average life expectancy, has improved significantly since 1999. For newly born women it has grown from 77.5 years of age to 81.1 years of age in 2013, while at the same time for men it grew from 66.8 years of age to 73.1 years of age (Central Statistical Office). However, this does not change the fact that, according to the reports by Eurostat, life expectancy in Poland is shorter than the average value of this measure for the EU countries (3.2 years shorter for men [13]. This might be connected with the fact that Polish citizens drink more strong alcohols and beer than an average European and far less wine. The percentage of smoking men and women gives our country respectively the 20th and 19th place in Europe.

The major causes of death in Poland in 2010 were cardiovascular disease (which was the cause of 46 % of deaths in 2010) and cancer, which caused the deaths of 24.5 % of Poles in the same period.

According to forecasts by Eurostat, Poland belongs to the fastest-ageing societies of the EU. At the end of 2014, there were 5.9 million people recorded in Poland aged 65 and over, which constituted 15.3 % of the population.

In such dramatic circumstances, a parallel lack of cooperation between basic health care and hospitals occurred. Due to financial problems, hospitals try to limit the number of admissions and basic health care doctors either very rarely issue referrals or because of much restricted personal, financial, and competence abilities transfer patients to hospitals. Hospitals are heavily in debt; in the first quarter of 2014, the sum of public hospital debts amounted to PLN 9.924 billion (Central Statistical Office).

## Chosen priorities in the scope of system organization and structure

The most important organizational problem of the Polish health care system seems to be the existence of the central and highly monopolistic insurance market of the public purse, namely the NHF. The existence of one institution that finances health care services, which requires extensive knowledge on the scope of health needs of communities from various regions of the country and also the knowledge on supply capacity of the health care sector, appears to be a utopian idea to implement if the system is organized in this way. The policies implemented by such a body are strongly influenced by various political groups in power. Moreover, the centralized role of the NHF is inefficient and is based on an annual cycle of public finance management or by establishing the limits of patients in a given specialization for a given year, which results in constant lengthening of queues to see specialists. Thus, the only way of achieving, from the public purse, the function of a competent representative of the society and its health needs is transferring such tasks to bodies of lower, local government [1], which in the present political situation, unfortunately remains only a theory.

Another important challenge that the Polish health care system is facing is to create coordinated health care, understood as a system taking care of the patient, multi-specialist diagnosing, and treatment and determining it with the proper cooperation of various medical institutions. However, the most important thing is to create such a system in which patients do not feel lost and the continuity of the care is secured for them. An example of organizing medical care in such a way was introduced on January 1, 2015, so-called oncological package, the main goal of which is to lead a patient quickly and efficiently through subsequent stages of cancer diagnosis and treatment.

The introduction of coordinated medical care requires, among others, improving the relationships between a GP and a specialist, in a way integrating these two medical care levels and implementing a jointly created medical standard.

For several years, a particularly important organizational problem of the Polish health care system is the lack of physicians, including specialists. As the reports by the WHO show (Global Health Observatory data repository [3]), Poland is a country with a very low ratio of physicians per 1000 inhabitants. In 2012, in Poland it was 2.22 while, for example, in Spain 4.89, Portugal 4.1, and Norway 4.28.

## Legal nature priorities

There have been numerous controversies in Polish legislation concerning the health sector; the results of these have been amendments and resolutions.

For the current Minister of Health adopted by Polish Parliament on September 14, 2015, the law on public health seems to be incomplete; the law needed amendments in the area of financing, strengthening the competences of the public health representative, including the procreation health area into the National Health Programme [10].

A consequence of the medicament reimbursement law (which came into effect in 2012), Poland will be supplied with previous generation medicaments, as the NHF establishes its reimbursement patterns on the basis of cheap medicaments. The price of medicaments can change from month to month, but Polish inhabitants are still in the vanguard of countries where expenditures on medicaments are highest in Europe (in 2013 they constituted as much as 21.6 % of all health expenditures [9]).

A very controversial issue in our country is asserting patient rights in courts. Since 1999 lots of patients have brought charges concerning medical errors. However, even presently, court proceedings take years and the compensation granted is disproportionately low with relation to errors made by physicians. The reason for this is on one hand the slowness of courts, and on the other hand an exaggerated solidarity in the medical fraternity, which makes it difficult to conduct a quality assessment of the action in question.

## Priorities in the scope of financing

The main source of money for the sector is the public purse, the NHF. Money that goes to it comes from the health insurance contribution paid by the inhabitants. However, recently more and more Polish citizens are employed on the basis of civil law contracts; in these contracts, for specific tasks and contracts of mandate, contributions are not paid. Thus, the group that pays health insurance contributions finances the health expenditures of those who do not pay them. Moreover, the NHF receives money that comes from contributions of such institutions as the Social Insurance Office (ZUS) and the Agricultural Social Insurance Fund (KRUS), which have a problem with collecting contributions themselves, not to mention transferring the money to the NHF.

The way the resources for health protection are divided and criteria of health services contracting are controversial, too. Service providers accuse the NHF of unsatisfactory pricing of services and the unrealistic or even unnecessary requirements with reference to staff and equipment.

## Priorities in the scope of health

The Ministry of Health made a decision about the necessity and even obligation to create regional health maps, and although the main goal of the project is improving the quality of managing the health care system resources through developing and popularizing prognostic tools (http://www.mz.gov.pl) [6], a lot of space is devoted in it to evaluation of demographic and epidemiologic trends. The maps are to be created for particular disease entities with the consideration of the existing infrastructure and resources. The picture of the needs in the scope of a given disease entity is created for the whole of the country, while a better idea would be to divide the country into particular regions and analyzing them carefully with a wide range of health needs in mind, considering various diseases that result from, for example, geographical, economic, or cultural conditions of these places.

With respect to the health situation of the country, a very important task of the national government and local governments seems to be a concern for the education of the younger generation in prevention in particular.

## Needs in the scope of quality

In order to take care of the patients that will not only ensure them treatment in accordance with laws of the medical profession but will also offer them access to novel technologies, latest therapies, modern medicaments and innovative ways of treatment, and proper resources are required, which, as it has already been presented, in Poland are very much limited. Another required element is qualified staff, emigrate in large numbers from Poland to other countries.

The second area of service quality is connected with their service side, which is the non-medical aspect of services. Standards in this area have much to be desired. Overcrowded hospital rooms, beds put in corridors, tasteless meals, out-of-date toilets, out-of-order lifts or lack of them, are unfortunately still the harsh reality of some Polish medical institutions.

## Conclusions

Polish health care is far from being ideal. In the rankings in comparison with other countries, also the neighboring ones, its picture is bad. Patients, physicians themselves, and politicians complain about it.

The present situation of the Polish health care system has been and still is influenced by numerous political, market, economic, and human factors. The changes that have been introduced still do not seem to fully include the superior role of the patient in the system.

Presently, the most important influence on the health care system seems to be economic and demographic factors. Not all values of indexes representing these areas of the current Polish sector are satisfactory, although while comparing them with previous years, which also had an impact on this sector, they show a good change of direction. For example, the mortality of infants is decreasing, the average life expectancy of Polish inhabitants is increasing, the quality of patient service and medical services are improving. For a few years, the Polish health care system has been recording numerous actions connected with implementation of modern information technology systems. The work aimed at placing the system in the teleinformatic area are going in different directions, the most important of which are: administrative side of hospital operation, supporting current work of physicians in the area of diagnosing, counseling and therapy and making data on patients and latest achievements of the world medicine available [7].

This all seems to be destroyed by the tremendous underfinancing of the sector, placing Poland in the group of European countries least financed from public resources. However, is money a way to solve all the sector problems? Or are the factual reasons for the unsatisfactory condition of the system, ethics and culture, put on the back burner [2]?
